# Association of Resilience, Organisational Support and Family Function With Reality Shock Among Chinese Newly Graduated Nurses: A Cross‐Sectional Study

**DOI:** 10.1155/jonm/2222949

**Published:** 2026-05-20

**Authors:** Ruiying Jia, Dandan Liang, Fang Chen, Meijuan Lan, Lan Zhou, Liying Wang

**Affiliations:** ^1^ Department of Nursing, The Second Affiliated Hospital of Zhejiang University School of Medicine, Hangzhou, China, z2hospital.com

**Keywords:** family function, nurses, organisational support, reality shock, resilience

## Abstract

**Aim:**

To describe the levels of reality shock, examine its relationships with resilience, organisational support and family function, and identify associated factors among newly graduated nurses.

**Background:**

Reality shock is a prevalent challenge for newly graduated nurses. However, evidence regarding its associations with resilience, organisational support and family function remains limited.

**Methods:**

A cross‐sectional study was conducted among 482 newly graduated nurses. Descriptive statistics, univariate analysis, Pearson correlation analysis and multiple linear regression analysis were performed.

**Results:**

A total of 482 newly graduated nurses completed the questionnaire. The mean reality shock score was 94.06 ± 22.47. Pearson correlation analysis showed that resilience, organisational support, and family function were significantly correlated with reality shock. In addition, educational background, average daily working hours and sleep duration were identified as significant associated factors.

**Conclusion:**

Newly graduated nurses experienced a moderate level of reality shock. Several factors, including educational background, working hours and sleep duration were significantly associated with reality shock. In particular, resilience, organisational support and family function, were negatively correlated with reality shock.

**Implications for Nursing Management:**

The findings suggest that resilience, organisational support and family function may be relevant considerations when supporting newly graduated nurses. Strategies aimed at enhancing resilience and promoting supportive organisational and family environments may be considered as part of efforts to address reality shock during the transition to clinical practice.

## 1. Background

The global nursing workforce is facing a critical shortage [1, 2], with high turnover among newly graduated nurses contributing substantially to workforce instability [3, 4]. Nearly 30% of newly graduated nurses leave their unit or position within the first year of practice [5]. Supporting their transition into professional roles is therefore essential for workforce retention.

A key challenge during this transition is reality shock [6], defined as the discrepancy between expectations formed during education and the realities encountered in clinical practice [7]. Newly graduated nurses often experience reduced confidence, difficulties in managing complex workloads, and insufficient professional support [8, 9]. Elevated levels of reality shock have been associated with weakened professional identity, impaired well‐being, delayed adaptation, increased adverse events, and reduced quality of care [10, 11]. Identifying factors that may mitigate reality shock is therefore of considerable importance [12].

Individual and environmental resources may play important roles in shaping reality shock. Resilience, defined as the ability to adapt to stressors [13], has been associated with better emotional regulation and coping in demanding clinical settings [14]. Although some studies have reported a negative association between resilience and transition shock [15], findings remain inconsistent [16]. Organisational support, which reflects the extent to which employees feel valued and supported by their organisation [17], has been linked to improved job satisfaction, reduced turnover intention, and enhanced work engagement [18–20]. Strengthening organisational support may facilitate the transition of newly graduated nurses and foster their professional development [21]. However, its specific relationship with reality shock among newly graduated nurses remains underexplored.

In addition, family function, as an important source of social support, may influence nurses’ adaptation during the transition period [22]. It refers to the capacity of a family system to provide emotional cohesion, adaptability, and material support [23]. Effective family functioning has been associated with improved emotional stability, stronger professional identity, and better overall well‐being [24–27]. Nevertheless, limited research has examined its role in relation to reality shock among newly graduated nurses.

Existing studies have primarily examined individual, organisational, or social factors in isolation. Few have simultaneously investigated resilience, organisational support, and family function within an integrated framework to explain reality shock among newly graduated nurses in China. Therefore, this study aimed to examine the associations of resilience, organisational support, and family function with reality shock among Chinese newly graduated nurses.

## 2. Methods

### 2.1. Study Design and Participants

A descriptive cross‐sectional survey using convenience sampling was conducted among newly graduated nurses in several public hospitals in Zhejiang Province, China, between September and October 2024. Participants were eligible if they (1) were registered nurses currently employed at the hospital; (2) had ≤ 1 year of work experience since graduation and (3) provided informed consent and agreed to participate voluntarily. Exclusion criteria included (1) nursing interns and external trainees and (2) individuals on leave due to illness, pregnancy or personal reasons. The sample size was calculated using formula *N* = 4*U* *α* ^2^ *S* ^2^/*δ* ^2^ [28]. Based on a presurvey (*S* = 0.52), with an allowable error of 0.1 and *α* = 0.05, the minimum required sample size was estimated to be 416. To account for potential invalid responses, 485 questionnaires were distributed. After excluding three incomplete or invalid responses, 482 valid questionnaires were included in the final analysis.

### 2.2. Measures

#### 2.2.1. Demographic Information

A self‐designed questionnaire was used to collect participants’ demographic and work‐related characteristics, including age, gender, only‐child status, educational background, department and average daily working hours, whether they were employed in the same hospital as their internship, and average sleep duration over the past month.

#### 2.2.2. The Reality Shock Scale

Reality shock was assessed using the Reality Shock Scale developed by Çiriş Yildiz et al. [6] and localised by Wu et al. [29]. The scale demonstrated good content validity during development and cross‐cultural adaptation. It consists of 41 items across four dimensions: relations and cooperation, professional knowledge, responsibilities, and performance. Each item is rated on a five‐point Likert scale ranging from 1 (never) to 5 (regularly), with higher scores indicating greater levels of reality shock. The Cronbach’s *α* in this study was 0.97.

#### 2.2.3. Connor‐Davidson Resilience Scale‐10 (CD‐RISC‐10)

Resilience was measured using the CD‐RISC‐10 [30], a shortened version of the original scale [13]. Each item is rated on a five‐point Likert scale from 1 (never) to 5 (always), with higher scores indicating greater resilience. The CD‐RISC‐10 has demonstrated good validity and reliability in previous studies [31]. The Cronbach’s *α* in this study was 0.96.

#### 2.2.4. The Nurses’ Survey of Perceived Organisational Support Scale (SPOS)

Organisational support was assessed using the nurses’ SPOS, originally developed by Eisenberger et al. [32] and revised by Zuo [33]. The scale includes 13 items across two dimensions: emotional support (items 1–10) and instrumental support (items 11–13). Each item is rated on a five‐point Likert scale ranging from 1 (strongly disagree) to 5 (strongly agree), with higher scores indicating greater organisational support. The scale has demonstrated good psychometric properties in previous studies [34, 35]. The Cronbach’s *α* in this study was 0.96.

#### 2.2.5. The Family Care Index Scale (APGAR)

Family function was measured using the Chinese version of the Family Care Index (APGAR) [23]. The scale comprises five items assessing adaptation, partnership, growth, affection and resolution. Each item is rated on a three‐point Likert scale (0 = almost never, 1 = sometimes and 2 = always), with total scores ranging from 0 to 10. Higher scores indicate better family functioning. Scores of 0–3 indicate severe dysfunction, 4–6 moderate dysfunction and 7–10 good family functioning. The Chinese version has demonstrated good reliability and validity [36, 37]. The Cronbach’s *α* in this study was 0.87.

### 2.3. Data Collection

Data were collected between September and October 2024 through an online survey administered via Questionnaire Star, a widely used professional survey platform in China. Each participant received a questionnaire along with an information sheet explaining the study purpose, voluntary participation, and anonymity. All responses were collected anonymously.

### 2.4. Statistical Analysis

Data were analysed using SPSS Version 21.0. Descriptive statistics (frequency, mean and standard deviation) were used to summarise participant characteristics and study variables. The Kolmogorov–Smirnov test was conducted to assess the normality of continuous variables. Independent samples *t*‐tests or one‐way analysis of variance (ANOVA) was conducted to compare differences in reality shock across subgroups. Pearson correlation analysis was used to examine relationships between variables. Multiple linear regression analysis was performed to identify factors associated with reality shock. Statistical significance was set at *p* < 0.05 (two‐tailed).

### 2.5. Ethical Considerations

This study was approved by the Ethics Committee of the Second Affiliated Hospital of Zhejiang University School of Medicine (Approval No: IR20240508). Participants completed the questionnaire anonymously. Participation was voluntary and anonymous. All participants provided electronic informed consent prior to participation.

## 3. Results

### 3.1. The Demographic Characteristics of the Participants

A total of 482 newly graduated nurses completed the questionnaire, including 427 females (88.6%) and 55 males (11.4%). Most participants were aged under 25 years (94.6%), were not only children (72.0%), and held a bachelor’s degree or below (95.2%). Regarding work conditions, 59.1% reported working 8.1–10 h per day, and more than 70% were not employed in the same hospital in which they had completed their clinical training. In terms of sleep patterns, 73.9% reported an average sleep duration of 6–8 h per day (Table 1).

**TABLE 1:** Characteristics of the participants (*n* = 482).

Variable	*N*	%
Age(years old)		
≤ 25	456	94.6
> 25	26	5.4
Gender		
Male	55	11.4
Female	427	88.6
Only child in the family		
Yes	135	28.0
No	347	72.0
Education background		
Bachelor’s or below	459	95.2
Master’s or above	23	4.8
Departments		
Internal medicine	143	29.7
Surgery	127	26.3
Obstetrics and gynaecology	8	1.7
Paediatrics	10	2.1
Emergency or intensive care unit	113	23.4
Operating room	31	6.4
Others	50	10.4
Average daily working hours		
≤ 8	122	25.3
8.1–10	285	59.1
> 10	75	15.6
Were employed in the same hospital as their internship		
Yes	134	27.8
No	348	72.2
Average sleep duration over the past month (h)		
< 6	111	23.0
6–8	356	73.9
> 8	15	3.1

*Note: N* = frequency, % = percentage, h = hours.

### 3.2. Scores of Study Variables

The mean reality shock score was 94.06 ± 22.47. The mean scores for resilience, organisational support and family function were 25.78 ± 7.85, 47.52 ± 9.12 and 6.93 ± 2.78, respectively (Table 2).

**TABLE 2:** Mean scores of the study variables (*n* = 482).

Variables	Total score range	M ± SD	Items scores
Reality Shock	61∼158	94.06 ± 22.47	2.29 ± 0.54
Relationships and cooperation	31∼78	45.16 ± 11.26	2.15 ± 0.54
Professional knowledge	14∼50	26.05 ± 7.74	2.36 ± 0.70
Responsibilities	8∼29	13.99 ± 4.76	2.33 ± 0.79
Performance	4∼14	8.86 ± 2.25	2.95 ± 0.75
Resilience	0∼40	25.78 ± 7.85	2.58 ± 0.78
Organizational support	13∼63	47.52 ± 9.12	3.65 ± 0.70
Emotional support	10∼48	35.21 ± 7.45	3.52 ± 0.75
Instrumental support	3∼15	12.31 ± 2.01	4.10 ± 0.67
Family function	0∼10	6.93 ± 2.78	1.39 ± 0.56
Adaptation	0∼2	1.23 ± 0.73	1.23 ± 0.73
Partnership	0∼2	1.23 ± 0.72	1.23 ± 0.72
Growth	0∼2	1.43 ± 0.66	1.43 ± 0.66
Affection	0∼2	1.55 ± 0.61	1.55 ± 0.61
Resolution	0∼2	1.47 ± 0.67	1.47 ± 0.67

*Note:* M = mean.

Abbreviation: SD, standard deviation.

### 3.3. Univariate Analysis of Reality Shock Among Newly Graduated Nurses

Univariate analyses indicated that reality shock differed significantly by educational background, department, average daily working hours and average sleep duration (all *p* < 0.05). Higher levels of reality shock were observed among nurses working more than 10 h per day compared with those working ≤ 8 or 8–10 h. Similarly, participants who reported less than 6 h of sleep per day experienced higher reality shock than those with longer sleep durations. Regarding departmental differences, nurses working in operating rooms, surgical units, and emergency or intensive care units reported higher levels of reality shock compared with those in paediatrics and other departments (Table 3).

**TABLE 3:** Univariate analysis of reality shock among newly graduated nurses (*n* = 482).

Variables	*N*	M ± SD	*t*/*F*	*p*
Age(years)				
≤ 25	456	94.00 ± 22.43	−0.246	0.805
> 25	26	95.12 ± 23.65		
Gender				
Male	55	97.71 ± 23.08	1.281	0.201
Female	427	93.59 ± 22.38		
Only child in the family				
Yes	135	93.89 ± 21.71	−0.103	0.918
No	347	94.12 ± 22.79		
Educational background				
Bachelor’s or below	459	93.54 ± 22.40	−2.289	0.023^∗^
Master’s or above	23	104.48 ± 21.81		
Departments				
Internal medicine	143	92.09 ± 21.12	3.109	0.005^∗^
Surgery	127	96.55 ± 23.55		
Obstetrics and gynaecology	8	83.75 ± 21.45		
Paediatrics	10	78.50 ± 9.93		
Emergency/intensive care unit	113	96.78 ± 23.22		
Operating room	31	101.23 ± 22.92		
Others	50	87.52 ± 20.34		
Average daily working hours				
≤ 8	122	82.61 ± 16.76	48.23	< 0.001^∗^
8.1–10	285	94.18 ± 21.03		
> 10	75	112.21 ± 23.99		
Were employed in the same hospital as their internship				
Yes	134	97.23 ± 20.38	1.929	0.054
No	348	92.84 ± 23.14		
Average sleep duration over the past month (h)				
< 6	111	106.95 ± 24.50	27.152	< 0.001^∗^
6–8	356	90.49 ± 20.40		
> 8	15	83.27 ± 17.75		

*Note: N* = frequency, M = mean. *t* = *t* test value, *F* = ANOVA value, h = hours.

Abbreviation: SD, standard deviation.

^∗^ *p* < 0.05.

### 3.4. Pearson’s Correlation Analysis

Pearson’s correlation analysis showed that reality shock was negatively correlated with resilience (*r* = −0.534, *p* < 0.01), organisational support (*r* = −0.603, *p* < 0.01) and family function (*r* = −0.467, *p* < 0.01). Resilience was positively correlated with organisational support (*r* = 0.592, *p* < 0.01) and family function (*r* = 0.503, *p* < 0.01). In addition, organisational support was positively associated with family function (*r* = 0.490, *p* < 0.01) (Table 4).

**TABLE 4:** Pearson’s correlation coefficients of the study variables (*n* = 482).

Variable	Reality shock	Resilience	Organizational support	Family function
Reality shock	1			
Resilience	−0.534^∗^	1		
Organizational support	−0.603^∗^	0.592^∗^	1	
Family function	−0.467^∗^	0.503^∗^	0.490^∗^	1

^∗^ *p* < 0.01.

### 3.5. Multivariable Linear Regression Analysis

Independent variables were assigned values according to the requirements of multiple linear regression, with dummy variables set for categorical variables, as shown in Table 5. All variance inflation factors were below 10, indicating no significant multicollinearity. Variables that were significant in the univariate analysis, together with resilience, organisational support and family function, were included in the regression model. The results showed that education background (*β* = 0.105, *p* = 0.002), average daily working hours (*β* = 0.189, *p* < 0.001), average sleep duration over the past month (*β* = −0.089, *p* = 0.012), resilience (*β* = −0.214, *p* < 0.001), organisational support (*β* = −0.325, *p* < 0.001) and family function (*β* = −0.130, *p* = 0.001) were significant predictors of reality shock. The model explained 48% of the variance (*F* = 37.981, *p* < 0.001). A Supporting stratified analysis was conducted by classifying departments into high‐intensity and general departments. The results were consistent with the primary model, and the association between department and reality shock was attenuated after adjusting for work‐related and psychosocial variables. Supporting Information 1 provides the stratified analysis by department intensity, with the corresponding results presented in Supporting Table 1. The primary regression model based on the original departmental categories is presented in Table 6.

**TABLE 5:** Assignment of independent variables.

Variables	Assignment criteria
Educational level	Bachelor’s or below = 1, Master’s or above = 2
Department	Internal medicine (H1 = 0, H2 = 0, H3 = 0, H4 = 0, H5 = 0, H6 = 0, H7 = 0), Surgery (H1 = 0, H2 = 1, H3 = 0, H4 = 0, H5 = 0, H6 = 0, H7 = 0), Obstetrics and gynaecology (H1 = 0, H2 = 0, H3 = 1, H4 = 0, H5 = 0, H6 = 0, H7 = 0), Paediatrics (H1 = 0, H2 = 0, H3 = 0, H4 = 1, H5 = 0, H6 = 0, H7 = 0), Emergency or Intensive care unit (H1 = 0, H2 = 0, H3 = 0, H4 = 0, H5 = 1, H6 = 0, H7 = 0), Operating room (H1 = 0, H2 = 0, H3 = 0, H4 = 0, H5 = 0, H6 = 1, H7 = 0), Others (H1 = 0, H2 = 0, H3 = 0, H4 = 0, H5 = 0, H6 = 0, H7 = 1)
Average daily working hours	< 8 h = 1, 8–10 h = 2, > 10 h = 3
Average sleep duration over the past month	< 6 h = 1, 6–8 h = 2, > 8 h = 3
Resilience	Initial value
Organisational support	Initial value
Family function	Initial value

*Note:* h = hours.

**TABLE 6:** Multivariable linear regression analysis.

Variables	*β*	*t*	*p*	Tolerance	VIF
(Constant)	—	20.116	< 0.001^∗^	—	—
Education background	0.105	3.139	0.002^∗^	0.974	1.027
Average daily working hours	0.189	5.057	< 0.001^∗^	0.774	1.292
Average sleep duration over the past month	−0.089	−2.531	0.012^∗^	0.875	1.143
Surgery	−0.004	−0.090	0.929	0.704	1.421
Obstetrics and gynaecology	−0.039	−1.145	0.253	0.949	1.054
Paediatrics	−0.050	−1.485	0.138	0.947	1.056
Emergency/Intensive care unit	−0.023	−0.594	0.553	0.692	1.446
Operating room	0.010	0.283	0.777	0.810	1.235
Others	−0.006	−0.169	0.866	0.812	1.232
Resilience	−0.214	−4.919	< 0.001^∗^	0.573	1.745
Organisational support	−0.325	−7.415	< 0.001^∗^	0.561	1.781
Family Function	−0.130	−3.220	0.001^∗^	0.666	1.503

*Note:* *R* ^2^ = 0.493; adjusted *R* ^2^ = 0.480; *F* = 37.981; *p* < 0.001.

Abbreviation: VIF, variance inflation factor.

^∗^ *p* < 0.05.

## 4. Discussion

This study identified a moderate level of reality shock (2.29 ± 0.54) among newly graduated nurses, consistent with previous findings [6]. Among the four dimensions, performance and professional knowledge scored the highest, indicating that these areas represent primary challenges in the transition to clinical practice. Reality shock is a common challenge for newly graduated nurses, as it undermines care quality and increases the risk of missed care [38]. Elevated levels of reality shock negatively impact nurses’ physical and mental health and compromise clinical outcomes [39]. Reality shock is also linked to weaker professional identity, delayed adaptation, and higher turnover intentions, thereby hindering the formation of a stable nursing workforce [15, 40]. Given these far‐reaching consequences, addressing this issue should be a priority.

The performance dimension reflects how a nurse’s work is perceived by others and underscores the challenges newly graduated nurses face due to insufficient feedback and support [6]. Nursing demands a broad skill set beyond core clinical competencies, yet new nurses often encounter unsustainable workloads, long hours, poor communication and limited recognition [41]. Such conditions can lead to emotional distress, especially when their efforts are overlooked or met with negative feedback [42]. The professional knowledge dimension assesses newly graduated nurses’ self‐perceived understanding, skills and competencies. However, a gap often exists between theoretical training and real‐world clinical demands, leaving them unprepared for practice [43]. Limited professional knowledge combined with inadequate experience frequently leads to confusion, stress and increased risk of errors [44, 45]. To mitigate these challenges, nursing managers should adopt a comprehensive approach addressing physical, psychological, and professional pressures. Recommended strategies include targeted training in communication and practical skills, timely recognition of performance, and the creation of a supportive work environment. Early identification of transition‐related difficulties and timely interventions can further ease adaptation, enhance team stability and ultimately improve patient care outcomes.

This study found a significant negative correlation between resilience and reality shock, consistent with prior research [46]. As a key psychological resource, resilience enhances optimism, perseverance and emotional stability, enabling newly graduated nurses to better manage stress and adapt to clinical challenges [47]. Resilient nurses recover faster, adjust learning strategies more flexibly and engage more actively in their work [48]. Additionally, resilience serves as an internal protective factor, helping nurses mitigate the negative effects of reality shock [15]. Similarly, organisational support was significantly negatively correlated with reality shock, indicating its role in promoting positive transition experiences. According to the Conservation of Resources Theory, organisational support restores emotional resources depleted during the transition to clinical practice [49]. It also fosters a sense of belonging, strengthens professional identity, and enhances role adaptation [50, 51]. Furthermore, a high level of organisational support helps nurses develop a clearer understanding of their professional value and effectively reduces the impact of reality shock [52]. This study found a significant negative correlation between family function and reality shock, highlighting family support as a key protective factor during the transition to professional nursing. A supportive family offers emotional security, practical guidance and a stable environment, which help reduce stress and enhance adaptability [53]. In such an environment, nurses are more likely to stay optimistic, regulate emotions, and use positive coping strategies to handle workplace challenges [54]. Conversely, poor family function may increase stress levels, making it difficult for nurses to balance family and work‐related conflicts [55]. These findings underscore the need to incorporate family support mechanisms—such as mentorship, peer groups and work‐life balance initiatives—into nurse transition programmes.

Multiple linear stepwise regression analysis revealed that educational background, daily working hours, average sleep duration, resilience, organisational support and family function were significantly associated with reality shock among newly graduated nurses. Notably, nurses with master’s degrees reported higher levels of reality shock than those with lower educational backgrounds, which contradicts previous findings [56]. Given that the present study did not directly assess factors that may underlie this association, such as clinical training hours, career expectations or department assignment mechanisms, the reasons for this difference remain unclear. Future research incorporating direct measures of these potential influencing factors is warranted to further clarify the relationship between educational level and reality shock. Longer working hours were associated with higher levels of reality shock, which aligns with prior research [57]. Extended working hours have been linked to increased burnout and job dissatisfaction, which may further complicate the transition from academic settings to clinical practice [58]. Similarly, shorter sleep duration was associated with higher levels of reality shock, which is consistent with previous research [56]. Sleep deprivation may exacerbate stress, impair cognitive functioning, reduce work efficiency and limit the capacity to adapt to demanding clinical environments [59, 60]. These findings suggest that work–rest balance may be an important consideration in supporting newly graduated nurses during the transition period. Approaches such as reasonable scheduling, adequate rest, and supportive management practices may help facilitate smoother adaptation to clinical roles. The Supporting stratified analysis further supported the findings of the primary regression model. After the department was operationalised as department intensity, its association with reality shock was no longer statistically significant. This finding suggests that the differences observed across departments in the univariate analysis may reflect variations in workload‐related factors rather than department classification itself. However, as these factors were not directly or comprehensively measured, this interpretation should be approached with caution. Future studies are warranted to further examine the role of departmental and work environment characteristics in shaping reality shock among newly graduated nurses.

The multiple linear stepwise regression analysis indicated that resilience was significantly associated with reality shock among newly graduated nurses, which is inconsistent with some previous findings [16]. While resilience is generally considered a protective factor in adapting to workplace challenges [61], its relationship with reality shock may vary across cultural, educational and organisational contexts. This inconsistency highlights the need for further research to clarify how resilience interacts with transition experiences and workplace demands, thereby informing more targeted support strategies for newly graduated nurses. Organisational support was also significantly associated with reality shock. Support from leadership, colleagues and institutional policies may provide important guidance, emotional reassurance and practical resources during the transition to clinical practice [62]. Such support may help enhance confidence, reduce stress and facilitate role adaptation [63], whereas insufficient support may be associated with feelings of isolation and increased reality shock [51]. These findings suggest that structured mentorship, clear communication and supportive leadership may be important considerations in facilitating the transition of newly graduated nurses. Similarly, family function was significantly associated with reality shock, indicating that family support may play an important role during this transitional period. Supportive family environments may provide emotional and financial stability, which can help newly graduated nurses cope with workplace stress [54, 64]. In contrast, poorer family function may be associated with increased stress and difficulties in role adaptation [24]. These findings suggest that family‐related factors may be considered when developing support strategies and transition programmes for newly graduated nurses.

## 5. Limitations

This study has several limitations that should be acknowledged. First, due to the cross‐sectional design, causal relationships between variables cannot be established. Longitudinal studies are needed to examine changes in reality shock over time. Second, this study did not collect detailed information on clinical training experiences, preservice preparation or career expectations. As a result, the underlying mechanisms explaining differences in reality shock, particularly across educational levels, could not be fully explored. Third, although department type was included in the analysis, residual confounding cannot be ruled out. For example, nurses with higher educational levels may be more likely to work in high‐intensity settings, which could partially explain the observed differences. Finally, convenience sampling was used, and participants were recruited from several hospitals in Zhejiang Province. In addition, the sample sizes across departments were uneven, which may have limited statistical power for subgroup analyses. Therefore, the generalisability of the findings may be limited. Future studies using more diverse and representative samples are recommended.

## 6. Implications for Nursing Management

Based on the findings of this study, resilience, organisational support and family function were significantly associated with reality shock among newly graduated nurses. These findings suggest that nursing managers may consider paying attention to these factors when supporting newly graduated nurses during their transition to clinical practice. First, as resilience was negatively associated with reality shock, nursing managers may consider assessing the practice readiness of newly graduated nurses in terms of professional knowledge and clinical skills and providing individualised guidance during the transition period. Interventions aimed at enhancing resilience, such as stress management training, mentoring programmes and psychological adjustment training, may be considered as part of supportive strategies to assist newly graduated nurses in coping with clinical challenges. Second, given that organisational support was negatively associated with reality shock, efforts to foster a supportive work environment and a positive organisational climate may be beneficial. Approaches such as providing constructive feedback, managerial support and opportunities for communication may be considered to support newly graduated nurses as they adjust to workplace demands. Third, as family function was also associated with reality shock, nursing managers may consider supporting newly graduated nurses in balancing work and family responsibilities where feasible. Flexible scheduling and supportive organisational policies may be explored as potential approaches to facilitate their transition to clinical practice. Finally, nursing managers may consider promoting a supportive and inclusive workplace atmosphere, maintaining open communication, and identifying newly graduated nurses who may require additional support. Incorporating assessments of reality shock, resilience, perceived organisational support and family function into routine evaluations may help inform targeted support strategies.

## 7. Conclusion

In conclusion, the participants experienced a moderate level of reality shock. Factors such as educational background, average daily working hours and average sleep duration were significantly associated with reality shock. In addition, reality shock was negatively correlated with resilience, organisational support and family function. As an important component of the nursing workforce, newly graduated nurses may encounter challenges during the transition to clinical practice. The findings of this study suggest that attention to individual, organisational and family‐related factors may be relevant when developing supportive strategies for this population. However, given the cross‐sectional design of this study, these findings should be interpreted as associations rather than causal relationships. Future research is warranted to further examine these relationships using longitudinal or experimental designs and to explore the underlying mechanisms of reality shock among newly graduated nurses.

## Author Contributions

Ruiying Jia: conceptualisation, methodology, formal analysis, investigation, writing–original draft and writing–review and editing. Dandan Liang: methodology, investigation, data curation, writing–original draft and writing–review and editing. Fang Chen: conceptualisation, investigation, writing–review and editing, project administration and funding acquisition. Meijuan Lan: data curation and writing–review and editing. Lan Zhou: writing–review and editing. Liying Wang: writing–review and editing.

## Funding

This work was supported by the Medical and Health Science Program of Zhejiang Province (grant numbers 2026782946).

## Disclosure

All intellectual content, study design, analysis, and conclusions are the sole responsibility of the authors.

## Conflicts of Interest

The authors declare no conflicts of interest.

## Supporting Information

Additional supporting information can be found online in the Supporting Information section.

## Supporting information


**Supporting Information** Supporting Information 1. Supporting stratified analysis using department intensity. This Supporting file presents the stratified analysis of reality shock according to department intensity among newly graduated nurses.

## Data Availability

The data that support the findings of this study are available from the corresponding author upon reasonable request.

## References

[1] Boniol M. , Kunjumen T. , Nair T. S. , Siyam A. , Campbell J. , and Diallo K. , The Global Health Workforce Stock and Distribution in 2020 and 2030: a Threat to Equity and ‘Universal’ Health Coverage?, BMJ Global Health. (2022) 7, no. 6, 10.1136/bmjgh-2022-009316. PMC9237893 35760437

[2] World Health Organization , Nursing and Midwifery, 2024, https://www.who.int/news-room/fact-sheets/detail/nursing-and-midwifery.

[3] Tamata A. T. and Mohammadnezhad M. , A Systematic Review Study on the Factors Affecting Shortage of Nursing Workforce in the Hospitals, Nursing Open. (2023) 10, no. 3, 1247–1257, 10.1002/nop2.1434. 36303066 PMC9912424

[4] Ulupinar S. and Aydogan Y. , New Graduate Nurses′ Satisfaction, Adaptation and Intention to Leave in Their First Year: a Descriptive Study, Journal of Nursing Management. (2021) 29, no. 6, 1830–1840, 10.1111/jonm.13296. 33639015

[5] Kovner C. T. , Djukic M. , Fatehi F. K. et al., Estimating and Preventing Hospital Internal Turnover of Newly Licensed Nurses: a Panel Survey, International Journal of Nursing Studies. (2016) 60, 251–262, 10.1016/j.ijnurstu.2016.05.003, 2-s2.0-84971475330. 27297385

[6] Çiriş Yildiz C. and Ergün Y. , Development and Psychometric Testing of the Reality Shock Scale for Newly Graduated Nurses, Journal of Advanced Nursing. (2021) 77, no. 12, 4950–4962, 10.1111/jan.15056. 34590733

[7] Kramer M. , Reality Shock: Why Nurses Leave Nursing, American Journal of Nursing. (1974) 75, no. 5, 10.1097/00000446-197505000-00041.

[8] Monaghan T. , A Critical Analysis of the Literature and Theoretical Perspectives on theory-practice Gap Amongst Newly Qualified Nurses Within the United Kingdom, Nurse Education Today. (2015) 35, no. 8, e1–e7. 10.1016/j.nedt.2015.03.006 25862073

[9] See E. C. W. , Koh S. S. L. , Baladram S. , and Shorey S. , Role Transition of Newly Graduated Nurses from Nursing Students to Registered Nurses: a Qualitative Systematic Review, Nurse Education Today. (2023) 121, 10.1016/j.nedt.2022.105702. 36577288

[10] Su Q. , Wu Y. , Yun B. , Zhang H. , She D. , and Han L. , The Mediating Effect of Clinical Teaching Behavior on Transition Shock and Career Identity Among New Nurses: a cross-sectional Study, Nurse Education Today. (2023) 125, 10.1016/j.nedt.2023.105780. 36963229

[11] Labrague L. J. , Relationship Between Transition Shock in Novice Emergency Room Nurses, Quality of Nursing Care, and Adverse Patient Events: the Mediating Role of Emotional Exhaustion, Australas Emerg Care. (2024) 27, no. 1, 9–14, 10.1016/j.auec.2023.07.001. 37442703

[12] Aldosari N. , Pryjmachuk S. , and Cooke H. , Newly Qualified Nurses′ Transition from Learning to Doing: a Scoping Review, International Journal of Nursing Studies. (2021) 113, 10.1016/j.ijnurstu.2020.103792. 33120135

[13] Connor K. M. and Davidson J. R. , Development of a New Resilience Scale: the Connor-Davidson Resilience Scale (CD-RISC), Depression and Anxiety. (2003) 18, no. 2, 76–82, 10.1002/da.10113, 2-s2.0-0141731320. 12964174

[14] Cooper A. L. , Brown J. A. , Rees C. S. , and Leslie G. D. , Nurse Resilience: a Concept Analysis, International Journal of Mental Health Nursing. (2020) 29, no. 4, 553–575, 10.1111/inm.12721. 32227411

[15] Li Y. R. , Liu J. Y. , Fang Y. , Shen X. , and Li S. W. , Novice Nurses′ Transition Shock and Professional Identity: the Chain Mediating Roles of self-efficacy and Resilience, Journal of Clinical Nursing. (2024) 33, no. 8, 3161–3171, 10.1111/jocn.17002. 38379345

[16] Kim S. O. and Kim J. S. , Association of Work Environment and Resilience with Transition Shock in Newly Licensed Nurses: a cross-sectional Study, Journal of Clinical Nursing. (2021) 30, no. 7-8, 1037–1045, 10.1111/jocn.15649. 33434340

[17] Rhoades L. and Eisenberger R. , Perceived Organizational Support: a Review of the Literature, Journal of Applied Psychology. (2002) 87, no. 4, 698–714, 10.1037/0021-9010.87.4.698, 2-s2.0-84880973434. 12184574

[18] Poghosyan L. , Ghaffari A. , Liu J. , and McHugh M. D. , Organizational Support for Nurse Practitioners in Primary Care and Workforce Outcomes, Nursing Research. (2020) 69, no. 4, 280–288, 10.1097/nnr.0000000000000425. 32058457 PMC9869754

[19] Çetin Aslan E. , Türkmen İ. , and Top M. , The Effect of macro-control and Organizational Support Perception on Nurses and Physicians Intention to Quit During the COVID-19 Pandemic, Journal of Nursing Scholarship. (2023) 55, no. 4, 843–852, 10.1111/jnu.12849. 36494753 PMC9877762

[20] Al-Hamdan Z. and Bani Issa H. , The Role of Organizational Support and self-efficacy on Work Engagement Among Registered Nurses in Jordan: a Descriptive Study, Journal of Nursing Management. (2022) 30, no. 7, 2154–2164, 10.1111/jonm.13456. 34415087

[21] Baharum H. , Ismail A. , McKenna L. , Mohamed Z. , Ibrahim R. , and Hassan N. H. , Success Factors in Adaptation of Newly Graduated Nurses: a Scoping Review, BMC Nursing. (2023) 22, no. 1, 10.1186/s12912-023-01300-1. PMC10111715 37069647

[22] Zhang H. , Jiang J. X. , Zhong M. H. et al., Career Adaptability of Newly Graduated Nurses at an Obstetrics and Gynaecology Hospital in China: a Qualitative Study, Journal of Nursing Management. (2022) 30, no. 6, 2046–2053, 10.1111/jonm.13661. 35506471

[23] Smilkstein G. , The Family APGAR: a Proposal for a Family Function Test and Its Use by Physicians, Journal of Family Practice. (1978) 6, no. 6, 1231–1239. 660126

[24] Bu T. , Peng C. , Zhang J. et al., Relationship Between Family Function and Anxiety Among Nurses During the COVID-19 Pandemic: a Mediating Role of Expressive Suppression, BMC Nursing. (2024) 23, no. 1, 10.1186/s12912-024-02167-6. PMC11287927 39075483

[25] Kim S. C. , Quiban C. , Sloan C. , and Montejano A. , Predictors of Poor Mental Health Among Nurses During COVID-19 Pandemic, Nursing Open. (2021) 8, no. 2, 900–907, 10.1002/nop2.697. 33570266 PMC7753542

[26] Zhang B. , Tao H. , Xie M. , Zhang J. , Zhang M. , and Zhang Y. , Factors Associated with Professional Identity Among ICU Nurses During COVID-19: a cross-sectional Study, Nursing Open. (2023) 10, no. 8, 5701–5710, 10.1002/nop2.1817. 37208994 PMC10333909

[27] Xu S. , Ju D. , Chen Y. et al., Analysis of the Correlation Between Clinical Nurses′ Professional Quality of Life and Family Care and Organizational Support, Frontiers in Public Health. (2023) 11, 10.3389/fpubh.2023.1108603. PMC9992405 36908408

[28] Ni P. , Chen J. L. , and Liu N. , The Sample Size Estimation in Quantitative Nursing Research, Chinese Journal of Nursing. (2010) 45, no. 04, 378–380.

[29] Wu M. , Wu D. , and Zhang X. , Translation and Psychometric Characteristics of the Reality Shock Scale for Newly Graduated Nurses, Journal of Nursing Science. (2023) 38, no. 12, 76–79.

[30] Campbell-Sills L. and Stein M. B. , Psychometric Analysis and Refinement of the Connor-davidson Resilience Scale (CD-RISC): Validation of a 10-item Measure of Resilience, Journal of Traumatic Stress. (2007) 20, no. 6, 1019–1028, 10.1002/jts.20271, 2-s2.0-38049017534. 18157881

[31] Wang L. , Shi Z. , Zhang Y. , and Zhang Z. , Psychometric Properties of the 10-item Connor-Davidson Resilience Scale in Chinese Earthquake Victims, Psychiatry Clin Neurosci. (2010) 64, no. 5, 499–504, 10.1111/j.1440-1819.2010.02130.x, 2-s2.0-77957351538. 20923429

[32] Eisenberger R. , Huntington R. , Hutchison S. , and Sowa D. , Perceived Organizational Support, Journal of Applied Psychology. (1986) 71, no. 3, 500–507, 10.1037/0021-9010.71.3.500, 2-s2.0-0000281443.

[33] Zuo H. , A Study on Relationship Between Organizational Support Emotion and Organizational Commitment of Nurses, 2009, Shanxi Medical University.

[34] Gu Z. H. , Qiu T. , Tian F. Q. , Yang S. H. , and Wu H. , Perceived Organizational Support Associated with Depressive Symptoms Among Petroleum Workers in China: a Cross-Sectional Study, Psychology Research and Behavior Management. (2020) 13, 97–104, 10.2147/PRBM.S232635. 32095086 PMC6995304

[35] Liu W. , Zhao S. , Shi L. et al., Workplace Violence, Job Satisfaction, Burnout, Perceived Organisational Support and Their Effects on Turnover Intention Among Chinese Nurses in Tertiary Hospitals: a cross-sectional Study, BMJ Open. (2018) 8, no. 6, 10.1136/bmjopen-2017-019525, 2-s2.0-85053117625. PMC6009508 29886440

[36] Huang Y. , Liu Y. , Wang Y. , and Liu D. , Family Function Fully Mediates the Relationship Between Social Support and Perinatal Depression in Rural Southwest China, BMC Psychiatry. (2021) 21, no. 1, 10.1186/s12888-021-03155-9. PMC7953569 33711987

[37] Wang B. , Yuan D. , Zhong X. , Yang F. , and Fu H. , Family Function, Anxiety and Depression in Adults with Disabilities: a Network Analysis, Frontiers in Public Health. (2023) 11, 10.3389/fpubh.2023.1181203. PMC10644035 38026317

[38] Labrague L. J. , Reality Shock in Newly Graduated Critical Care Nurses and Its Association with Missed Nursing Care: the Mediating Role of Caring Ability, Intensive and Critical Care Nursing. (2024) 80. 10.1016/j.iccn.2023.103554 37837835

[39] Zhang X. , Huang H. , Du H. , and Li D. , Mitigating the Impact of Emotional Exhaustion Among Young Intensive Care Unit Nurses: a Structural Equation Model Based on the Conservation of Resources Theory, Nursing in Critical Care. (2025) 30, no. 5, 10.1111/nicc.13266. 39865532

[40] Zhou L. , Kachie Tetgoum A. D. , Quansah P. E. , and Owusu-Marfo J. , Assessing the Effect of Nursing Stress Factors on Turnover Intention Among Newly Recruited Nurses in Hospitals in China, Nursing Open. (2022) 9, no. 6, 2697–2709, 10.1002/nop2.969. 34190432 PMC9584492

[41] Kisanuki N. , Takase M. , and Yamamoto M. , Unveiling the Challenges Encountered by Newly Graduated Nurses During Night Shifts: a Descriptive Qualitative Study, International Journal of Nursing Science. (2024) 11, no. 4, 447–456, 10.1016/j.ijnss.2024.08.009. PMC11740304 39830920

[42] Najafi B. and Nasiri A. , Concerns of Novice Nurses in the First Year of Work: a Qualitative Content Analysis, SAGE Open Nurs. (2024) 10, 10.1177/23779608241300019. PMC11638982 39676904

[43] Han P. , Duan X. , Wang L. , Zhu X. , and Jiang J. , Stress and Coping Experience in Nurse Residency Programs for New Graduate Nurses: a Qualitative meta-synthesis, Frontiers in Public Health. (2022) 10, 10.3389/fpubh.2022.979626. PMC9530278 36203675

[44] Labrague L. J. and McEnroe-Petitte D. M. , Job Stress in New Nurses During the Transition Period: an Integrative Review, International Nursing Review. (2018) 65, no. 4, 491–504, 10.1111/inr.12425, 2-s2.0-85058210560. 29266201

[45] Narbona-Gálvez Á. , García-Iglesias J. J. , Ayuso-Murillo D. et al., Stress in Novice Nurses in New Work Environments: a Systematic Review, Frontiers in Public Health. (2024) 12, 10.3389/fpubh.2024.1463751. PMC11557553 39540096

[46] Cao X. , Li J. , and Gong S. , The Relationships of Both Transition Shock, Empathy, Resilience and Coping Strategies with Professional Quality of Life in Newly Graduated Nurses, BMC Nursing. (2021) 20, no. 1, 10.1186/s12912-021-00589-0. PMC8062214 33888101

[47] Lin Y. E. , Lin C. T. , Hu M. L. , Tzeng S. , and Chien L. Y. , The Relationships Among Perceived Stress, Resilience, Sleep Quality and first-month Retention of Newly Employed Nurses: a cross-sectional Survey, Nursing Open. (2023) 10, no. 6, 4004–4012, 10.1002/nop2.1659. 36813734 PMC10170957

[48] Cabrera-Aguilar E. , Zevallos-Francia M. , Morales-García M. et al., Resilience and Stress as Predictors of Work Engagement: the Mediating Role of self-efficacy in Nurses, Front Psychiatry. (2023) 14, 10.3389/fpsyt.2023.1202048. PMC10464840 37649562

[49] Zhang Z. , Wang T. , Zhao Y. , and Shi X. , Transition Shock Experience of Newly Graduated Nurses: a Qualitative Study, Contemporary Nurse. (2025) 61, no. 1, 21–32, 10.1080/10376178.2024.2432636. 39607884

[50] Zeng Z. , Wang X. , Bi H. et al., Factors that Influence Perceived Organizational Support for Emotional Labor of Chinese Medical Personnel in Hubei, Frontiers in Psychology. (2021) 12, 10.3389/fpsyg.2021.684830. PMC8222904 34177739

[51] Ren Y. , Li G. , Pu D. et al., The Relationship Between Perceived Organizational Support and Burnout in Newly Graduated Nurses from Southwest China: the Chain Mediating Roles of Psychological Capital and Work Engagement, BMC Nursing. (2024) 23, no. 1, 10.1186/s12912-024-02386-x. PMC11459715 39379880

[52] Cockerham M. , Beier M. E. , Branson S. , and Boss L. , Nurse Adaptability and Post-traumatic Stress Disorder Symptoms During the COVID-19 Pandemic: the Effects of Family and Perceived Organizational Support, Frontiers in Psychology. (2021) 12, 10.3389/fpsyg.2021.749763. PMC8934423 35317265

[53] Liu X. , Dong W. , Zhang J. et al., The Underlying Mechanisms of Family Function on Anxiety Among Nurses During the Public Health Emergency, Scientific Reports. (2025) 15, no. 1, 10.1038/s41598-025-91435-9. PMC11868614 40016260

[54] Zeng W. , Fang Q. , Wang C. et al., Perceived Family Function and Associated Predictors in Nurses: a Cross-Sectional Study, Front Psychiatry. (2022) 13, 10.3389/fpsyt.2022.904581. PMC9251054 35795026

[55] Yao X. , Wen S. , Song Z. , Wang J. , Shen Y. , and Huang X. , Work-Family Conflict Categories and Support Strategies for Married Female Nurses: a Latent Profile Analysis, Frontiers in Public Health. (2024) 12, 10.3389/fpubh.2024.1324147. PMC10958783 38525344

[56] Yao L. , Chen J. , Zhao Q. et al., Level and Influencing Factors of Transition Shock Among New Nurses in China: a Multicenter cross-sectional Study, Health Science Reports. (2023) 6, no. 12, 10.1002/hsr2.1758. PMC10726909 38111741

[57] Zhou S. , Ma J. , Fan S. et al., The Mediating Effect of Job Embeddedness on the Nursing Work Environment and Transition Shock Among New Nurses: a cross-sectional Study, Nurse Education in Practice. (2024) 78, 10.1016/j.nepr.2024.104034. 38924870

[58] Shah M. K. , Gandrakota N. , Cimiotti J. P. , Ghose N. , Moore M. , and Ali M. K. , Prevalence of and Factors Associated with Nurse Burnout in the US, JAMA Network Open. (2021) 4, no. 2, 10.1001/jamanetworkopen.2020.36469. PMC7862989 33538823

[59] Park E. , Lee H. Y. , and Park C. S. , Association Between Sleep Quality and Nurse Productivity Among Korean Clinical Nurses, Journal of Nursing Management. (2018) 26, no. 8, 1051–1058, 10.1111/jonm.12634, 2-s2.0-85054559929. 29855101

[60] Veal B. M. , Mu C. X. , Small B. J. , and Lee S. , Subjective Cognitive Abilities Correlate with Poor Sleep Among day-shift and night-shift Nurses, Journal of Sleep Research. (2021) 30, no. 5, 10.1111/jsr.13359. 33987895

[61] Aryuwat P. , Asp M. , Lövenmark A. , Radabutr M. , and Holmgren J. , An Integrative Review of Resilience Among Nursing Students in the Context of Nursing Education, Nursing Open. (2023) 10, no. 5, 2793–2818, 10.1002/nop2.1559. 36564896 PMC10077422

[62] Hussein R. , Salamonson Y. , Everett B. , Hu W. , and Ramjan L. M. , Good Clinical Support Transforms the Experience of New Graduates and Promotes Quality Care: a Qualitative Study, Journal of Nursing Management. (2019) 27, no. 8, 1809–1817, 10.1111/jonm.12880. 31556168

[63] Soco C. , Simonovich S. D. , Dillon D. , Bishop-Royse J. , and Lattner C. , Communication, Leadership and Organizational Support Facilitate Successful Transition into Practice for Nurse Practitioners in the Emergency Department, J Am Assoc Nurse Pract. (2020) 33, no. 12, 1156–1165, 10.1097/jxx.0000000000000500. 32890050

[64] Tselebis A. , Lekka D. , Sikaras C. et al., Insomnia, Perceived Stress, and Family Support Among Nursing Staff During the Pandemic Crisis, Healthcare (Basel). (2020) 8, no. 4, 10.3390/healthcare8040434. PMC7712233 33114662

