# Supplementary material for: Association of Resilience, Organisational Support and Family Function With Reality Shock Among Chinese Newly Graduated Nurses: A Cross‐Sectional Study
Source: J Nurs Manag. 2026 May 20;2026:2222949. doi: 10.1155/jonm/2222949 (PMC13189478; doi:10.1155/jonm/2222949)
Supplement: Supplementary file 1 — Supporting Information Supporting Information 1. Supporting stratified analysis using department intensity. This Supporting file presents the stratified analysis of reality shock according to department intensity among newly graduated nurses. [file JONM-2026-2222949-s001.docx]

**Supplementary stratified analysis using department intensity**

To further examine the robustness of the regression results and clarify the role of department, a supplementary stratified analysis was conducted by replacing the original department categories with a binary variable representing department intensity (high-intensity vs general departments). The results showed that department intensity was not significantly associated with reality shock after adjusting for education background, average daily working hours, average sleep duration, resilience, organizational support, and family function (*β* = 0.006, *P* = 0.851). In contrast, education background (*β* = 0.104, *P* = 0.002), average daily working hours (*β* = 0.191, *P* < 0.001), average sleep duration (*β* = −0.093, *P* = 0.008), resilience (*β* = −0.212, *P* < 0.001), perceived organizational support (*β* = −0.328, *P* < 0.001), and family function (*β* = −0.132, *P* = 0.001) remained significantly associated with reality shock. The model explained 48.1% of the variance in reality shock (adjusted *R^2^* = 0.481, *F* = 64.708, *P* < 0.001), and no multicollinearity was detected among the independent variables.

Supplementary Table 1 Supplementary multiple linear regression analysis using reclassified department categories (high-intensity vs. general departments).

| Variables | *β* | *t* | *P* | Tolerance | *VIF* |
| --- | --- | --- | --- | --- | --- |
| （Constant） | - | 18.712 | <0.001^*^ | - | - |
| Education background | 0.104 | 3.137 | 0.002^*^ | 0.981 | 1.019 |
| Average daily working hours | 0.191 | 5.248 | <0.001^*^ | 0.812 | 1.232 |
| Average sleep duration over the past month | -0.093 | -2.659 | 0.008^*^ | 0.884 | 1.131 |
| Department intensity | 0.006 | 0.188 | 0.851 | 0.911 | 1.098 |
| Resilience | -0.212 | -4.914 | <0.001^*^ | 0.582 | 1.719 |
| Organizational support | -0.328 | -7.556 | <0.001^*^ | 0.573 | 1.745 |
| Family Function | -0.132 | -3.300 | 0.001^*^ | 0.672 | 1.487 |

*Note:* high-intensity departments: emergency/intensive care units and operating rooms; general departments: internal medicine, surgery, obstetrics and gynaecology, paediatrics, and other
